# Challenges in Risk Stratification of Solid Atypical Mixed Echogenicity Thyroid Nodules

**DOI:** 10.17925/EE.2023.20.1.2

**Published:** 2023-11-09

**Authors:** Evana Valenzuela-Scheker, David N Bimston, Hubert Golingan, Allan Golding, R Mack Harrell

**Affiliations:** 1. Department of Endocrine Surgery, Memorial Healthcare System, Hollywood, FL, USA; 2. Department of Internal Medicine, Mount Sinai Hospital, Miami Beach, FL, USA

**Keywords:** Papillary thyroid cancer, risk stratification, thyroid neoplasms, thyroid nodule, ultrasound, solid atypical mixed echogenicity thyroid nodules

## Abstract

**Background**: To determine the prevalence and risk of malignancy (ROM) in solid atypical mixed echogenicity thyroid nodules (SAMENs) with sonographic patterns not classifiable by the 2015 American Thyroid Association Ultrasound Risk Stratification System (NC ATA). **Methods**: We searched our prospectively collected endocrine surgery thyroid nodule (TN) database, with particular attention to those solid nodules that were NC ATA. An algorithm assigned each into one of the five ATA risk groups per the 2015 American Thyroid Association Ultrasound Risk Stratification System (ATA USRSS). TNs that the algorithm could not assign to a risk group were deemed NC ATA and were subsequently analyzed. Additionally, we categorized this group using an algorithm based on the 2017 American College of Radiology Thyroid Imaging Reporting and Data System (ACR-TIRADS). We were specifically interested in the characteristics that resulted in non-classification by the 2015 ATA USRSS and the fine needle aspiration biopsy (FNAB) cytology and surgical pathology results from the group. **Results**: We evaluated data from 5,040 nodules, of which 1,772 had surgical pathology. There were 150 solid nodules not classified by 2015 ATA USRSS, all of which demonstrated atypical features along with iso-, hetero-, hyper-and mixed echogenicity (solid atypical mixed echogenicity nodules-SAMENs). Sixty of these nodules were excised and sent for surgical pathology, while 90 were followed without surgical excision. Out of the 90 that did not undergo surgery, 82 underwent FNAB with cytologic evaluation. Of our 150 SAMENs, 40 were malignant by surgical histology and six were likely malignant by cytology (total SAMEN ROM without noninvasive follicular thyroid neoplasm with papillary-l ike nuclear features 31%). The most common sonographic pattern present in our SAMEN group consisted of an isoechoic solid component with microcalcifications (28/40–70% of all excised malignant nodules). In our excised malignant SAMENs, 50% demonstrated follicular-patterned neoplastic architecture while 48% displayed papillary architecture. **Conclusion**: Our study demonstrates that SAMENs with at least one suspicious sonographic feature: including (1) microcalcifications; (2) irregular or other suspicious margins,;opulation, and a higher ROM (31%) than the intermediate-risk group of the 2015 ATA USRSS (10–20%).

In the USA, neck ultrasound (US) identifies thyroid nodules (TNs) in 30–50% of adult patients.^1,2^ Given that the risk of malignancy (ROM) for all combined thyroid nodule types ranges from 5% to 15%,^2,3^ current guidelines recommend US of the neck initially, to identify suspected TNs.^4^ Neck US is used to localize TNs, facilitate risk stratification, clarify the necessity for fine-needle aspiration biopsy (FNAB) and to evaluate the neck for metastatic lymph nodes and tumour invasiveness.

In 2015, the American Thyroid Association (ATA) created a new version of their guidelines that stratified TNs into five risk groups based on sonographic pattern, with recommendations for FNAB based on size and appearance on US.^4^ The 2015 ATA Ultrasound Risk Stratification System (USRSS) emphasized nodule risk stratification based on sonographic pattern rather than isolated sonographic features^5,6^ on the grounds that sonographic patterns have better diagnostic performance and reliability than individual sonographic features.^6^

Although the 2015 ATA USRSS simplifies risk stratification by using pictorial representations of several common sonographic patterns, there are ‘gap patterns’ that are seen in ‘atypical’ nodules, which are not addressed in this important work.^7^ The ATA USRSS ‘low suspicion’ pattern includes nodules with iso-or hyperechoic solid components, but without suspicious US features. These include microcalcifications, irregular or invasive margins, and a taller-than-wide shape. When atypical cystic or non-hypoechoic solid nodules exhibit suspicious US features, these technically remove them from the ATA USRSS benign, very low risk or low risk categories and render them ‘not classified’ (NC ATA) by ATA USRSS. Several researchers have found that nodules with these patterns constitute a subgroup at higher risk of malignancy (ROM).^7–14^ A recent meta-analysis by Kwon et al. examining findings from 16 studies of NC ATA nodules demonstrates that these nodules largely fall into two groups: (1) cystic atypical nodules (CANs); and (2) solid atypical mixed echogenicity nodules (SAMENs).^15^ We chose to examine our ATA NC thyroid nodule experience using our endocrine surgical database, with a particular emphasis on solid ATA NC nodules, which we refer to as SAMENs.

## Methods

Our practice is a high-volume endocrine surgical clinic specializing in thyroid, parathyroid and adrenal disorders that often require surgical intervention. After institutional review board approval Memorial Healthcare system MHS.2020.092 (Reference# 006854), we analyzed prospectively collected US characteristics, cytology and surgical histology data (with patient identifiers removed). These data had been entered into our endocrine surgical practice database between January 2014 and December 2019. Proprietary algorithms were used to risk stratify patients according to the 2015 ATA USRSS and 2017 American College of Radiology Thyroid Imaging Reporting and Data System (ACR TI-RADS) criteria.^16^

All USs were performed by the three Endocrine Certification in Neck Ultrasonography accredited imaging endocrinologist authors of this paper, and prospective data entry into the database was accomplished by these same physicians. All surgery was performed by our Memorial Health System endocrine surgeons, led by author DNB.

**Figure 1:**
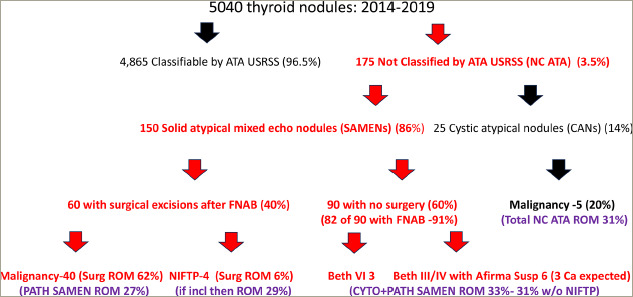
Study population breakdown

One hundred seventy-five of our 5,040 nodules (3.5%) were not classifiable by the 2015 ATA USRSS algorithm (*Figure 1*). Twenty-five of these nodules (14%) were spongiform or >75% cystic with hypoechoic or anechoic echo architecture accompanied by atypical features (cystic atypical nodules [CANs]) and these nodules were excluded from consideration in this ‘solid’ architecture investigation. The remaining 150 solid nodules (86% of the NC ATA nodules evaluated) were either isoechoic, heteroechoic, hyperechoic or mixed echogenicity, while exhibiting additional atypical US features; and therefore were categorized as SAMENs. Sixty of the 150 SAMENs (40%) underwent surgical excision and surgical pathologic evaluation and 82 of the remaining 90 nodules underwent FNAB with cytologic evaluation (91%).

In this report, a nodule's predominant composition was recorded as a quartile percentage (i.e.<25%, 25–50%, 50–75% or >75% cystic). For the purposes of this research, we elected to term all nodules with less than 75% cystic structure as ‘solid’. For entry into the SAMEN group of the study, the solid component of any <75% cystic nodule had to demonstrate iso-, hyper-, hetero-or mixed echogenicity, with at least one atypical feature. Spongiform nodules or nodules with cystic composition of 75% or more were classified as cystic. If they exhibited atypical features, they were considered CANs. In practice, SAMENs plus CANs equal ATA NC.

*ATA USRSS = American Thyroid Association Ultrasound Risk Stratification System; CAN = cystic atypical nodules; CYTO = cytology; FNAB = fine-needle aspiration biopsy; NC ATA = not classifiable by the 2015 American Thyroid Association Ultrasound Risk Stratification System; NIFTP = noninvasive follicular thyroid neoplasms with papillary-like nuclear features; PATH = pathology; ROM = risk of malignancy; SAMEN = solid atypical mixed echogenicity thyroid nodule. We studied 5,040 patients with thyroid nodules prospectively entered into our endocrine surgical database over six years from 2014 to 2019. One hundred and seventy-five of these (3.5%) were unclassifiable by the ATA USRSS guideline. Twenty-five of them were greater than or equal to 75% cystic with atypical features and 150 were <75% cystic with a significant solid portion that was iso-, hyper-, heteroechoic or of solid atypical mixed echogenicity nodules (SAMENs). Sixty of the SAMEN nodules underwent surgical removal, while 91% of the remaining 90 nodules underwent fine-needle aspiration biopsy for cytologic diagnosis. Best estimate risk of malignancy was 31% in the 150 SAMEN nodules, with four noninvasive follicular thyroid neoplasm with papillary-like nuclear features considered benign.*

Our atypical feature database fields included margin characteristics, calcification types and presence of taller-than-wide shape in axial (transverse) view. Nodule echogenicity was categorized as hypo-, iso-, hetero-, hyperechoic or mixed based on the nodule's appearance in comparison with the surrounding thyroid tissue and adjacent strap muscles.

Eighty-two of the 90 non-surgical SAMENs underwent FNAB and cytological analysis (cytology performed by an independent cytopathology laboratory) based on the Bethesda System for Reporting Thyroid Cytopathology.^17^

Sixty SAMENs were surgically excised. All surgical pathology analysis was performed by qualified pathologists in the Memorial Healthcare System. In our reporting, noninvasive follicular thyroid neoplasms with papillary-l ike nuclear features (NIFTP’s) were reported separately from thyroid cancer because, after 2016, these neoplasms were no longer considered to be cancer (our study ranged from 2014–2019).^18^ In an attempt to further clarify the risk of malignancy (ROM), molecular testing was performed in 12 of 40 cancers (30%) with Veracyte's Afirma Gene Expression Classifier (GEC) or Gene Sequencing Classifier (GSC) classifier (AFIRMA) for nodules with indeterminate (Bethesda III and IV) cytology. Eleven of 12 results were suspicious for malignancy. Mutational analysis was performed in 17 of 40 cancers, with an even split between classical papillary lineage^9^ and follicular lineage^8^ fusions and mutations.

## Results

Our database included 5,040 nodules from a patient population that was 17% male and 83% female. The median age for our thyroid nodule patients was 50.1 years. One hundred and seventy-five of our 5,040 nodules were NC ATA by ATA USRSS (175 out of 5,040 or 3.5%). Twenty-five of these unclassified nodules (14%) were equal to or greater than 75% CANs and these were excluded from further analysis because we chose to focus on the large majority of the NC ATA nodules that were atypical with clearly distinguishable solid components (SAMENs) (150 out of 175; 86%). All of these SAMENs were compositionally <75% cystic with solid portions exhibiting either isoechoic, heteroechoic, hyperechoic or mixed echogenicity. The great majority of our SAMENs (126/150 or 84%) were less than 25% cystic. In the 24 SAMENs with between 25% and 75% cystic composition, the significant solid portion of the nodule was assessed for echogenicity and atypical features.

### Non-surgical SAMEN group

Ninety of the 150 nodules with SAMENs did not undergo surgical excision, either because cytology was benign or the patient chose not to return for further evaluation.

The atypical US features that distinguished our non-surgical SAMENs from ATA USRSS low risk isoechoic nodules were: microcalcifications (41/90; 46%) and suspicious margins (including lobulated, irregular, infiltrative and indistinct border descriptors) (40/90; 44%) and taller-than-wide geometry in the transverse US plane (21/90; 23%). SAMEN solid portions were 72% isoechoic (66/90), 18% heteroechoic (15/90), 8% mixed echogenicity (7/90) and 2% hyperechoic (2/90); therefore, nodules in the surgical SAMEN cohort were almost exclusively isoechoic.

In the non-surgical SAMEN group, 91% (82 of 90) of nodules underwent FNAB. Cytologic evaluation revealed 66 nodules with Bethesda II benign or favour benign cytology (73%). Ten nodules had Bethesda III changes (11%) including five with suspicious AFIRMA testing, one of which carried a *BRAF* V600E mutation. There were four nodules with Bethesda IV changes (4%) including one with suspicious AFIRMA. Finally, there were two nodules (2%) with malignant cytology. In summary, based on FNAB results, there were three nodules out of the non-surgical 90 that had a high likelihood (>70%) of being thyroid cancer based on cytology or mutational analysis and five more Bethesda III and IV nodules with suspicious AFIRMA molecular testing (50% ROM for AFIRMA suspicious result). Therefore, for SAMEN ROM calculation purposes, we made an FNAB-based assumption that there were six malignancies in the non-surgical group of 90 (2 Bethesda VI cytologic results, one *BRAF* V600E mutation, and five Bethesda III/IV cytologic results with a 50% risk of malignancy based on uspicious AFIRMA GEC or GSC for a total of 5.5 malignancies, which we rounded up to six prior to ROM calculation).

### Surgical SAMEN group

The surgical SAMEN cohort almost exclusively contained isoechoic nodules (56/60; 93%) with 2 hetero-and 2 hyperechoic nodules. Like the non-surgical SAMEN group, these nodules averaged 2.4 cm in greatest dimension.

**Figure 2:**
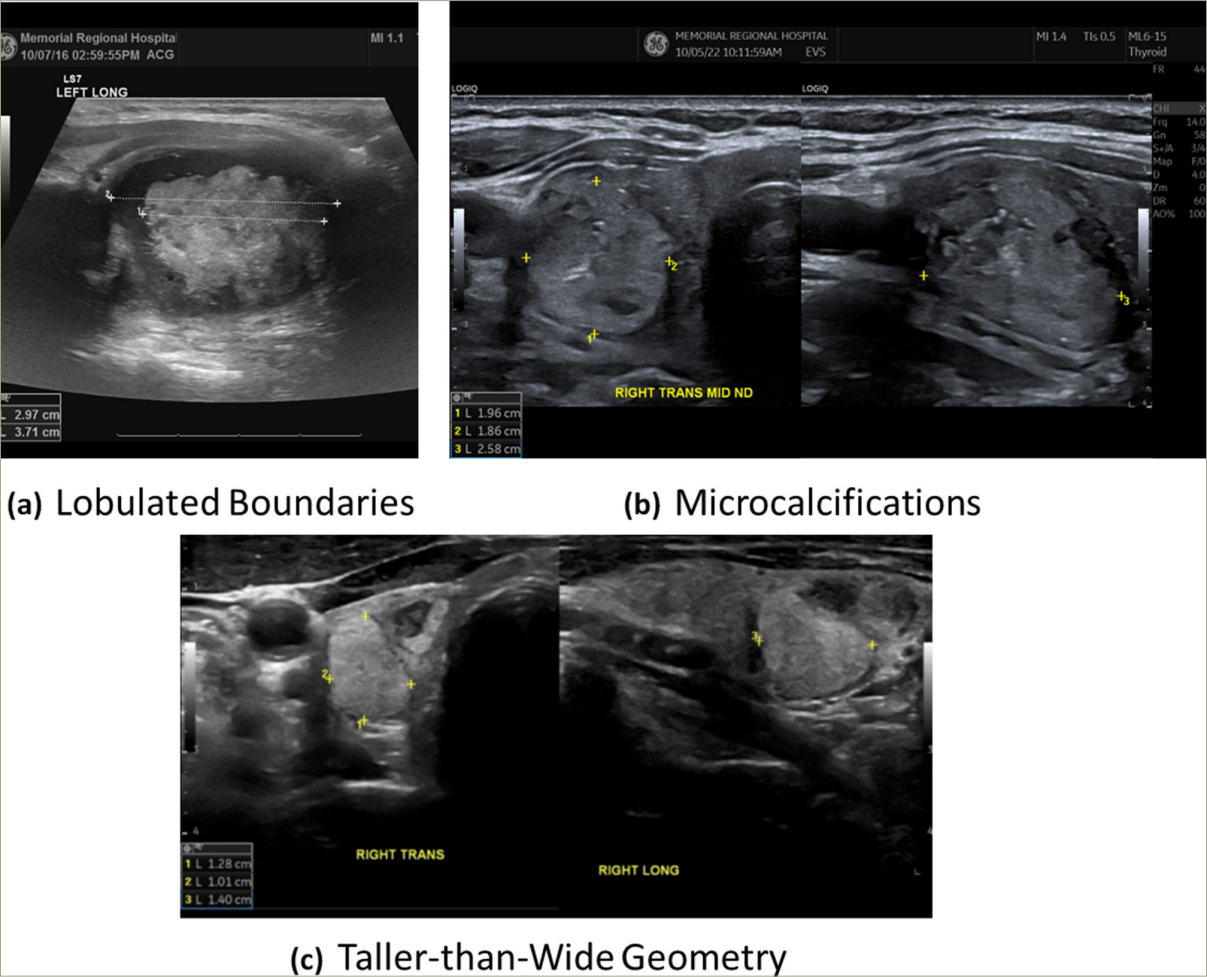
SAMEN US patterns

Surgical SAMENs were distinguished from ATA USRSS low risk isoechoic nodules by virtue of suspicious nodule margins (35/60; 58%), microcalcifications (31/60; 52%) and taller-than-wide US geometry in the transverse US view (10/60; 17%) (see *Figure 2*).

Fifty-four of the 60 surgical SAMEN patients (90%) underwent presurgical US-guided FNAB. Forty-three of the 60 (72%) had findings ranging from cellular atypia (Bethesda III) to frankly malignant cytology (Bethesda VI) and these riskier nodules were heavily concentrated in the 40 SAMENs with surgical pathology-documented malignancy (34/40 cancers; 85% with Bethesda III-VI cytology).

Surgical pathologic findings in this group showed 40 unequivocal cancers (67%) and four NIFTPs (7%). Twenty of the 40 malignancies were of follicular lineage (50%), with 19 classified as papillary thyroid cancers (48%) and a single Hurthle cell carcinoma (2%). The four NIFTPs in the surgical SAMEN group were not counted as malignancies. The most common echogenicity and atypical feature combination seen in our 40 excised thyroid cancers was isoechoic with microcalcifications (28/40; 70%) (see *Figure 2c*).

#### ATA USRSS versus ACR TI-RADS

All nodules in our database were also stratified using an algorithm based on the 2017 ACR TI-RADS guidelines.

Non-surgical SAMENs averaged 2.4 cm in greatest diameter, with 20 of the 90 nodules (23%) measuring less than 1.5 cm and therefore not meeting the isoechoic low risk nodule size criteria for FNAB by the 2015 ATA USRSS guidelines. The ACR TI-RADS guidelines recommend FNAB for TR4 and TR5 category nodules when their largest diameters equal or exceed 1.5 and 1.0 cm, respectively.^4^ Fifteen of the 90 non-surgical SAMENs (17%) would not have been biopsied by 2017 ACR TI-RADS criteria (because their size was less than 1.5 cm for those classified as ACR TI-RADS 4 or less than 1.0 cm for those classified as ACR TI-RADS 5).

In our surgical SAMEN group (n=60), there were 77% (46 of 60 nodules) TI-RADS 4 nodules and 23% (14/60 nodules) TI-RADS 5 category TNs. Per ACR TI-RADS criteria, 13 out of 60 (22%) of our surgically treated SAMENs should not have undergone FNAB and the exact same 13 of 60 (22%) would not have had FNAB recommended by ATA USRSS. These nodules consisted of 11 thyroid cancers and 2 NIFTPs.

*(a): A thick, irregular, hypoechoic capsule surrounding an isoechoic/hyperechoic lobulated classical papillary thyroid cancer in a 34-year-old male; (b) an isoechoic microcalcified (white oval) irregular-bordered follicular-variant of papillary thyroid cancer in a 76-year-old female; (c) a taller-than-wide isoechoic nodule in a 67-year-old male with classical papillary thyroid cancer.*

### Discussion

In our surgical practice, NC ATA nodules constituted 175 out of 5,040 nodules (3.5%) that we evaluated over 6 years. The vast majority of our NC ATA nodules were solid (SAMENs) (150/175; 86%) with the rest of the group consisting of cystic atypical nodules (CANs) (25/175; 14%).

The NC ATA nodules addressed by this study are best characterized as SAMENs. These difficult-to-classify nodules contain a distinctive solid portion (nodule is <75% cystic by definition) that is not hypoechoic (i.e. iso-, hetero-, hyper-and mixed echogenicity) with associated atypical features that include microcalcifications, suspicious borders and taller-than-wide geometry in the transverse US plane (see *Figure 2*).

#### Prevalence of NC ATA nodules in our endocrine surgical practice

Our overall NC ATA (SAMENs + CANs/total nodules) prevalence of 3.5% is lower than the pooled prevalence of Kwon et al. in their 2023 meta-analysis (7.8%),^15^ but similar to the prevalence reported by Ha et al. 1.8%,^14^ Gao et al. 2.7%^11^ and Yoon et al. 3.4%.^19^

#### Risk of malignancy in SAMENs

We found an unexpectedly high risk of malignancy (ROM 31%) in SAMENs when compared with the uncomplicated isoechoic (low-suspicion) group (ROM 5–10%) established by the 2015 ATA USRSS guidelines. Our ROM was calculated using our total number of cancers detected by surgical pathology (40) and cytology (6) divided by our total number of US-detected SAMENs during the six-year study period (46/150; 31%). We believe that this estimate is reasonably accurate given that 92% of our non-surgical group underwent FNAB with Bethesda System for Reporting Thyroid Cytopathology cancer risk assessment. Our estimate of 31% is close to the single-centre pooled estimate of 24.8% calculated by Kwon et al. in their meta-analysis^15^ and identical to the 31% calculated by Peng et al. in 2020.^20^

#### Risk associated with microcalcification

The appearance of microcalcifications increases the ROM for any solid nodule.^7^ Lauria-Pantano et al. demonstrated that ATA unclassified microcalcified TNs (NC ATA) had a seven-fold higher ROM by cytology than ‘very low suspicion’ nodules (OR=7.2 [CI=2.44–21.24], p<0.001].^7^ In one Korean study, when NC ATA TNs normally categorized as low risk were reclassified as intermediate suspicion nodules, the diagnostic sensitivity of FNAB increased to 99%.^9^

Prior to the publication of the 2015 ATA USRSS, Seo et al.^13^ noted a high ROM in isoechoic TNs with at least one suspicious US feature, although no isolated US feature was predictive of malignancy in isoechoic nodules. Additionally, Ahmadi et al.^10^ and Yoon et al.^12^ reported the most common sonographic pattern in their unclassified TNs (NC ATA) was an isoechoic solid component with microcalcifications, a finding corroborated by our surgical SAMEN data. This pattern is the most common pattern found in our surgically-excised malignant nodules (27/40; 68%). Although iso-, hyper-, hetero-and mixed echogenicity are US features often associated with benignity,^21–23^ the addition of at least one suspicious US feature appears to confer considerably higher ROM in isoechoic nodules, especially if that feature is microcalcification.

#### SAMENs: A mix of follicular and papillary cancer US and pathologic features

Ultrasonographically, SAMENs combine the microcalcifications and suspicious boundaries of papillary lineage thyroid cancers, with the brighter echogenicity of follicular lineage tumours to form hybrid patterned nodules with mixed papillary and follicular pathology and mutational findings.

Twenty of the 40 malignancies in our surgical SAMEN cohort were of follicular lineage (50%), with 19 classified as classical papillary thyroid cancers (48%) and a single Hurthle cell carcinoma (2%). There were four NIFTPs (also follicular architecture) in the surgical SAMEN group that were not counted as cancers. Although we only performed 17 mutational panels in our surgical SAMEN cohort of 60, it is interesting to note that mutations were evenly split into papillary and follicular camps. There were six *BRAF* V600E mutations, two RET PTC combinations and one ETV6 mutation (nine total), all associated with classical papillary cancers. There were five *NRAS* mutations, two atypical *BRAF* mutations (not *BRAF* V600E), one *HRAS* and one *KRAS* mutation (eight in total), genetic changes predominantly associated with follicular cancer.

#### Use of ATA USRSS and ACR TI-RADS in risky SAMENs

Similar to Gao et al., our results indicate that the ACR TI-RADS and the ATA USRSS both struggle to predict malignancy in SAMENs.^11^ ACR TI-RADS risk stratifies for different nodular US features including taller-than-wide geometry, punctate echogenic foci (i.e. microcalcifications), and irregular borders.^24^ In theory, ACR TI-RADS should suggest FNAB in SAMENs of sufficient size (1 cm in size for TR 5, and 1.5 cm for TR 4). However, the current ACR TI-RADS version still did not recommend FNAB in 28 of our 150 combined cohort SAMEN nodules (19%), where the documented ROM is 31%.

If our SAMENs were incorrectly classified into the isoechoic ATA guideline low risk group, the ATA USRSS performs slightly worse than ACR TI-RADS with no recommendation for FNAB in 33 of our combined SAMEN cohort of 150 nodules (22% no FNAB). This recommendation problem occurs because low risk nodules must be 1.5 cm or greater to receive an FNA recommendation by ATA USRSS. Thus, both the ACR TI-RADS and ATA USRSS risk stratification systems perform suboptimally when asked to categorize a higher risk nodule cohort like our SAMENs group.

#### Limitations of this investigation

There are several limitations of our study. Since the frequency of the SAMEN US pattern is relatively low in our highly selected endocrine surgery patient population (3.5%), the applicability of our data to a general endocrinology practice thyroid nodule referral base is uncertain.

Additionally, since we did not perform surgery on our entire SAMEN population, our risk of malignancy estimate is just a best estimate ROM. However, the additional strength of the cytologic data (91% FNAB rate in our 90-patient nonsurgical SAMEN group) suggests that a ROM in the 31% range is accurate, and compares favourably to the 16 study meta-analysis of Kwon et al. with a pooled single-cente ROM of 24.8% (confidence interval 13.0–28.7).^15^

### Conclusions

NC ATA SAMENs are mostly isoechoic nodules that also exhibit atypical features more commonly seen in classical papillary thyroid cancer including: microcalcifications, irregular boundaries and (3) taller than wide geometry (*Figure 2*). Both the 2015 ATA USRSS and the 2017 ACR TI-RADS systems under-recommend FNAB in our SAMEN study population. Based on our data and the findings of others, this group has a higher ROM (31%) than previously acknowledged. The ROM in our SAMEN cohort is higher than that assigned to the ATA USRSS intermediate suspicion group (10–20%) and, in the authors’ opinion, these nodules should be approached with at least as much diagnostic fervor as hypoechoic nodules of similar size. The expert panel members of the ATA USRSS and ACR TI-RADS guidelines should consider new FNAB criteria for this unique subset of thyroid nodules with particular attention to those between 1.0 and 1.5 cm in greatest dimension.
